# The cyclical lower extremity exercise for Parkinson’s trial (CYCLE): methodology for a randomized controlled trial

**DOI:** 10.1186/s12883-015-0313-5

**Published:** 2015-04-24

**Authors:** Anson B Rosenfeldt, Matthew Rasanow, Amanda L Penko, Erik B Beall, Jay L Alberts

**Affiliations:** Department of Biomedical Engineering, Cleveland Clinic, 9500 Euclid Avenue, Cleveland, OH 44195 USA; Imaging Institute, Cleveland Clinic, 9500 Euclid Avenue, Cleveland, OH 44195 USA; Cleveland FES Center, L Stokes Cleveland VA Medical Center, 10701 East Boulevard, Cleveland, OH 44106 USA; Center for Neurological Restoration, Cleveland Clinic, 9500 Euclid Ave., Cleveland, OH 44195 USA

**Keywords:** Parkinson’s disease, Magnetic resonance imaging, Forced exercise, Randomized clinical trial, Exercise, Methodology

## Abstract

**Background:**

Motor and non-motor impairments affect quality of life in individuals with Parkinson’s disease. Our preliminary research indicates that forced exercise cycling, a mode of exercise in which a participant’s voluntary rate of exercise is augmented on a stationary cycle, results in global improvements in the cardinal symptoms of Parkinson’s disease. The objective of the *Cyc*lical *L*ower Extremity *E*xercise (CYCLE) trial for Parkinson’s disease is to determine the effects of forced exercise cycling on motor and non-motor performance when compared to voluntary rate cycling and a non-exercise control group. Additionally, we plan to identify any associated changes in neural activity determined by functional magnetic resonance imaging.

**Methods/Design:**

A total of 100 individuals with mild to moderate idiopathic Parkinson’s disease will participate in a single-center, parallel-group, rater-blind study. Participants will be randomized 2:2:1 into a forced exercise, voluntary exercise, or no-exercise control group, respectively. Both exercise groups will cycle 3 times per week for 8 weeks at identical aerobic intensities for 40 minutes, but participants in the forced exercise group will cycle 30% faster than their voluntary rate by means of an augmented motorized bicycle. Neuroimaging, clinical, and biomechanical assessments of motor and non-motor performance will be made at baseline both ‘on’ and ‘off’ medication, after four weeks of exercise (midpoint), end of treatment, 4 weeks after end of treatment, and 8 weeks after end of treatment.

**Discussion:**

CYCLE trial will play a critical role in determining the effectiveness of two different types of aerobic exercise, forced and voluntary, on motor and non-motor performance in individuals with Parkinson’s disease. Additionally, the coupling of clinical, biomechanical, and neuroimaging outcomes has the potential to provide insight into mechanisms underlying change in function as a result of exercise.

**Trial registration:**

Clinicaltrials.gov registration number NCT01636297.

## Background

Parkinson’s disease (PD) is a chronic, progressive neurological disorder that affects an estimated 4 million individuals worldwide [1]. Individuals diagnosed with PD typically experience progressive deficits in motor and non-motor functions which contribute to diminished quality of life, cognitive impairments, fatigue, mood disorders, and anxiety [2-5]. Unfortunately, there is no treatment that can modify the disease process itself. Current therapies include symptom management through medical and surgical interventions; however, these treatments are expensive and can cause adverse side effects. Therefore, the development of a low-cost, non-invasive treatment that can improve symptoms of PD and improve quality of life would be valuable.

It has been well-established that exercise in healthy adults plays a role in decreasing the incidence of cardiovascular, metabolic, and musculoskeletal conditions, as well as preserving cognitive function and preventing dementia and depression [6]. The concept of ‘exercise is medicine’ for PD, is supported by evidence that regular exercise may delay the onset of PD symptoms [7], and can improve motor scores, balance, and quality of gait in those with the disease [8]. Nevertheless, there is still much debate over what type of exercise should be prescribed. A recent Cochrane Review notes that due to small sample sizes, methodological flaws, and a wide range of exercise interventions, there is insufficient evidence to recommend one mode of exercise over another [9].

Aerobic exercise has been shown to reduce inflammation, suppress oxidative stress, and stabilize calcium homeostasis, which all promote brain health [10]. Looking specifically at the role of aerobic exercise in neurological conditions, it may induce neuroplastic changes in the central nervous system (CNS) through the release of neurotrophic factors, which are capable of signaling neurons to survive, differentiate, and grow [11]. Animal models of PD support the importance of aerobic exercise in the release of neurotransmitters and neurotrophic factors, specifically brain derived neurotrophic factor and glial cell derived neurotrophic factor, which have been associated with positively impacting cognition and motor function in animal models of PD [12-15].

Previous studies suggests that exercise interventions must be intense and continuous to elicit benefits on PD symptoms [6,16]. However, individuals with PD typically experience varying levels of rigidity and bradykinesia, which may limit their ability to complete continuous, high-intensity exercise [17-19]. To address these issues, a novel mode of aerobic exercise termed forced exercise (FE) was developed to aid individuals with neurological deficits to achieve and maintain a high cadence pedaling rate. Initially, the FE paradigm was implemented using a tandem stationary cycle where the trainer on the front of the tandem bicycle pedaled between 80-90 rpms, and the individual with PD on the back of the bike was forced to pedal at the same rate due to the pedals on a tandem bike being mechanically linked by the bike chain [20,21]. In a preliminary study completed by Alberts and colleagues, motor outcomes were examined after individuals with PD completed 8 weeks of cycling using either the FE approach or voluntary exercise (VE), where individuals cycled on a stationary bike at a self-selected cadence [20,21]. The Unified Parkinson’s Disease Rating Scale (UPDRS) [22] motor subscale score for individuals randomized into the FE group improved 41% for rigidity, 38% for tremor, and 28% for bradykinesia. Additionally, biomechanical analysis of grasping force and torque indicated that FE, but not VE, resulted in an improvement in the coordination and control of grip and load forces during the performance of a bimanual object manipulation task [20,21]. Functional connectivity magnetic resonance imaging (fcMRI) was conducted during the following conditions: 1) on PD medication, 2) off PD medication, and 3) off medication after a single bout of FE. Results demonstrated that FE and PD medication produce similar brain connectivity responses, and likely share similar underlying mechanisms [23]. An improvement in upper extremity motor control processes following a lower extremity exercise intervention coupled with the change in fcMRI indicate that FE is altering brain structure and potentially function in some manner. However, the specific mechanism and identification of the phenotype of PD patients that could benefit from FE is unknown.

Not all studies examining high intensity exercise with PD have had such promising results. In a 2013 study, Qutubuddin and colleagues randomized 23 individuals with PD into a FE and no-exercise control group [24]. After 16 FE sessions, they found no difference between groups in quality of life or motor outcomes, including the UPDRS motor assessment [24]. A significant limitation of their study was that they did not use an active FE intervention. Rather, they used a cycle that could be programmed to move the pedals at a selected speed, however, the participants did not have to actively contribute to the pedaling action. Hence, it was passive high-rate exercise as opposed to FE as we have previously published which requires active participation of the patient. Additionally, participant heart rate (HR), cadence, and power were not reported in the results; therefore, it is not possible to know how much the participants were contributing or if they were experienced an elevated HR that was close to an aerobic zone. Shulman, et al. reported that low-intensity treadmill training produced changes in a 50-ft fast pace ambulation test and 6 minute walk times that were not seen in high-intensity treadmill training, and neither group displayed changes in UPDRS scores [25]. Notably, the low-intensity group received 50 minutes of treadmill training, while the high-intensity group received only 30 minutes; therefore, it is possible that time was an important factor in gait changes.

The *Cyc*lical *L*ower Extremity *E*xercise for Parkinson’s (CYCLE) Trial is designed to test the hypothesis that FE and VE, while both aerobic, produce different clinical outcomes as a result of differential effects on the CNS. Specifically, the aims of the CYCLE trial are: 1) To determine the effects of FE and VE on motor function in individuals with PD; 2) To determine the effects of FE and VE on non-motor function in individuals with PD; and 3) To determine the pattern of magnetic resonance imaging (MRI) activation associated with FE and VE. It is hypothesized that FE will elicit significantly greater improvements in clinical and biomechanical measures of motor and non-motor performance when compared to voluntary and no-exercise control groups. Further, it is hypothesized that FE will elicit increased cerebral blood flow and cortical and subcortical activation following exercise, while voluntary and no-exercise groups will result in no change in the pattern or extent of activation as seen on neuroimaging.

## Methods and design

### Study design

**Figure 1:**
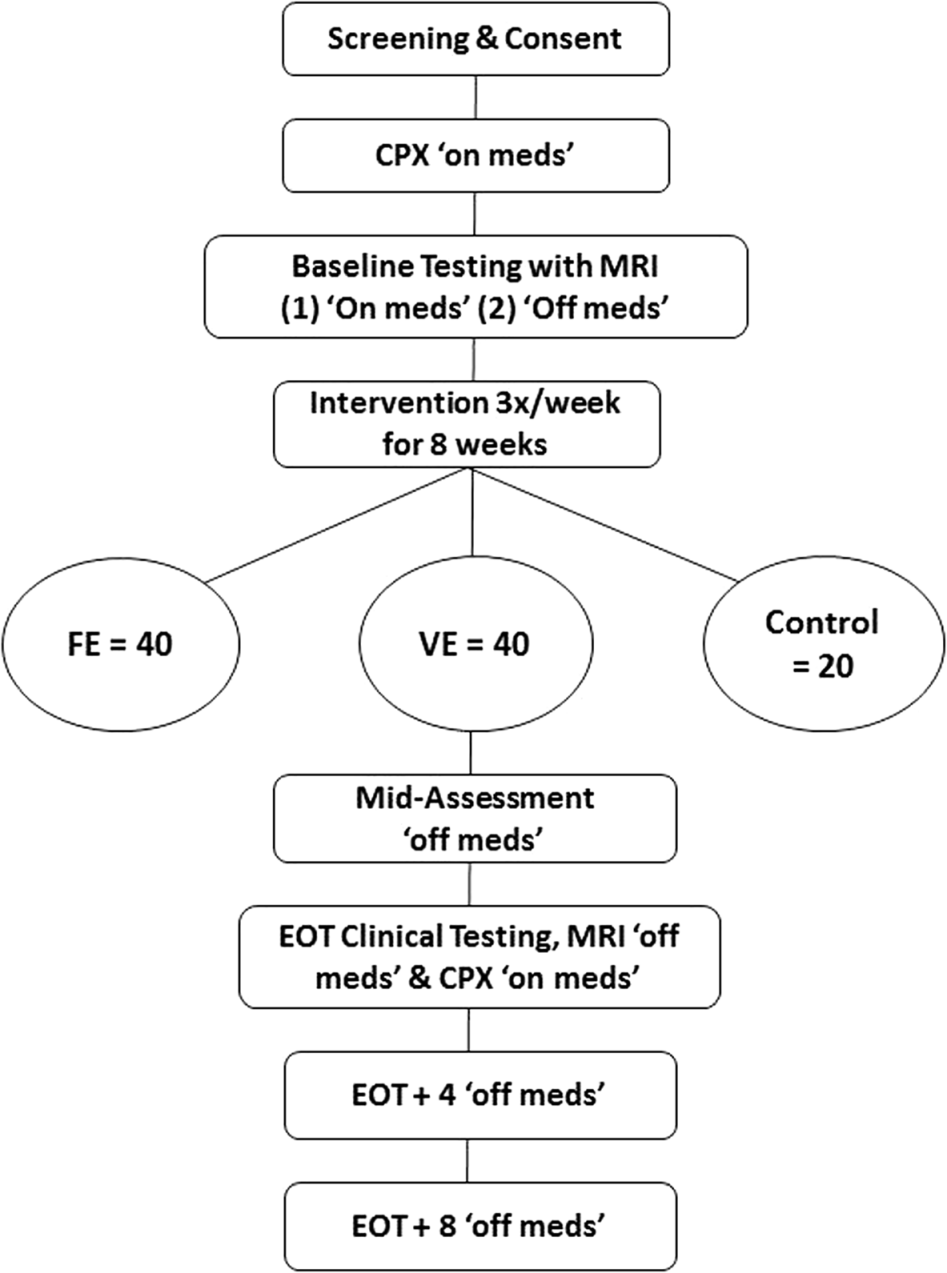
Study flow diagram outlining the 18 week enrollment.

This single center, prospective, rater-blind, three arm clinical trial will investigate the efficacy of a FE paradigm on motor and non-motor symptoms in individuals with PD. A total of 100 individuals with mild to moderate idiopathic PD will be enrolled. CYCLE Trial protocol is approved by the Cleveland Clinic Institutional Review Board and all participants will sign an informed consent prior to beginning the study. Written informed consent was obtained for any photographs that have been taken for publication. A schematic overview of the enrollment, testing, and intervention is shown in Figure 1.

### Power analysis

For Aim 1, the UPDRS motor score will be the primary outcome. Our power analysis was calculated based on UPDRS motor data from our previous project comparing FE and VE interventions on UPDRS ratings. We observed a mean (95% confidence interval (CI)) reduction of -5.3 (-18.0, 7.4) in the FE group and 0.4 (-15.3, 16.1) in the VE group from baseline to end of treatment (EOT) at 8 weeks. This corresponds to a difference (improvement, reduction) in means (95% CI) of -4.9 (-20.6, 10.8), standard deviation (SD) of difference = 8.0, P = 0.004. Assuming similar variability for the current proposal, with N = 40 subjects per exercise group, we will be able to detect differences of 5.0 or more between groups on UPDRS with 90% power at the 0.05 significance level, adjusting for 3 group comparisons at each of EOT evaluations. No published data exists for outcomes in Aim 2 or 3 with respect to exercise and PD. In general, with 40 subjects per exercise group, power will be 99 percent to detect differences of 1.2 SDs or more between the groups. For Aims 1-2, comparisons at particular time points power is 90% to detect differences of 1.3 SD, applying a Bonferroni correction for analyzing time points (2 post-intervention times) individually. All calculations assume Tukey adjustment for comparing the 3 groups.

### Subject enrollment

Participants will be recruited via patient education groups, study literature placement in exam rooms, patient chart reviews by research personnel, and in-service lectures at Cleveland Clinic affiliate and non-affiliate hospitals in the Cleveland-metro area.

Primary inclusion criteria for CYCLE Trial include: clinical diagnosis of idiopathic PD, between 30 and 75 years of age, not currently engaged in physical therapy for their PD or another interventional clinical study, Hoehn and Yahr stage II-III while on antiparkinsonian medication. Primary exclusion criteria include: presence of dementia, previous stroke, any medical or musculoskeletal contraindications to exercise, and existing cardiorespiratory disease as determined by American Heart Association/American College of Sports Medicine exercise pre-participation questionnaire [26,27].

### Cardiopulmonary exercise testing

Following completion of initial screening, participants will undergo cardiopulmonary exercise testing (CPX). CPX protocol is administered by an exercise physiologist from the preventative cardiology department of the Cleveland Clinic. Testing will be completed on a Lode cycle ergometer using MedGraphics CardiO_2_/CP system with Breeze software. The participant will be instructed to take his/her PD medication as prescribed on the day of testing. A 12-lead electrocardiogram will be assessed prior to exercise, continuously during exercise, and during exercise recovery. Participants will exercise at an initial load of 25 Watts, increasing by 25 Watts every two minutes until 100 Watts, and then increasing by 50 Watts every two minutes until the American College of Sports Medicine’s Guidelines for Exercise Testing criteria for test termination are reached [28]. The participant’s peak volume of oxygen uptake (VO_2_) will be calculated as the highest 30 second average of VO_2_ during the CPX test. A Cleveland Clinic cardiologist will interpret the results of the test, and those with normal responses to exercise will be randomized while those with abnormal responses will be advised for further medical work up.

### Randomization

Participants will be randomized 2:2:1 into FE, VE, or no-exercise control group. The uneven allocation of subjects was selected due to the promising results of the preliminary data by Alberts, et al. indicating there is a benefit to cardiovascular exercise that would not been seen in a control group [20,21]. Enrollment will total 18 weeks: two weeks allotted for baseline evaluations, 8 weeks of exercise intervention or control period, and 8 weeks for a follow up period in which the participant is instructed to return to their baseline level of physical activity.

### Voluntary exercise intervention group

Subjects randomized into the VE group will participate in 3 weekly exercise sessions over the course of 8 weeks for a total of 24 sessions. Exercise sessions will consist of a 5-minute warm up, a 40-minute main exercise set, and a 5-minute cool down on a standard stationary bicycle. All sessions will be completed under the guidance of an exercise physiologist. For participants who are deconditioned upon study enrollment, rest breaks will be allowed for 2 minutes every 10 minutes during the 40-minute main exercise set. Participants will be asked to pedal at a self-selected pace, and will be encouraged to achieve their target HR range, which will be calculated from their resting and maximal HR during the CPX test using the Karvonen formula at 60-80% of their max exertion [29]. HR, cadence, and power will be recorded every 5 minutes, and the rate of perceived exertion will be recorded every 10 minutes. Supervising exercise trainers and study personnel will be certified in Basic Cardiac Life Support. If a participant exhibits signs of cardiac distress or hemodynamic compromise, the exercise session will be stopped immediately and the on-call physician will be paged to the exercise laboratory. Following completion of the 24 session exercise intervention, participants will be instructed to resume pre-enrollment activity levels.

### Forced exercise intervention group

Participants in the FE group will receive a dose-matched intervention compared with the VE group. While our preliminary trials involved tandem cycling, in an attempt to make the intervention more feasible in a clinical setting, we have designed a stationary, motorized bike to augment self-selected cadence. The clear difference between the cycling groups is that while the VE group will use a standard stationary bike, participants in the FE group will be using a standard stationary bicycle that has been retrofitted with a motor to augment pedaling rate. In order to ensure that the participant is actively contributing to the pedaling motion, the algorithm used to control the assisted-cycle is responsive to pedal rate, the torque exerted on the pedals by the participant, and the torque produced by the motor during exercise. Based on our preliminary work with FE and PD, the cadence will be set at a rate that is approximately 30% greater than the individual’s self-selected pace during the CPX test [21]. It is important to note that although the participant will be cycling at an augmented rate, it is an active, not passive, activity. Power, cadence, HR, and rating of perceived exertion will be recorded at the same time intervals as the VE group.

### No exercise control group

Participants in the no-exercise control group will be instructed to maintain their pre-enrollment level of activity throughout the duration of their study enrollment. The study’s exercise physiologist will conduct weekly phone calls to ensure adherence, itemize participants’ activity levels, and monitor current medication regimes.

### Testing and outcome measures

Clinical tests, biomechanical tests, and MRI results will be compared at the following time points: baseline, midpoint, EOT, EOT + 4 weeks, and EOT + 8 weeks as depicted in Table 1. All testing will be completed in the ‘off med’ state, where the participant will be ask to abstain from their antiparkinsonian medication after 8pm the night preceding testing. The exception is baseline testing, which will be conducted over 2 days, one day in the ‘on med’ state and the other in the ‘off med’ state (randomized). The Research Electronic Data Capture (REDCap©) Database, a secure electronic database, will be used to record and store data.

**Table 1:** **Summary of outcome measures**

**Assessment**	Baseline*	Baseline*	**Mid-point**	**EOT**	**EOT + 4 weeks**	**EOT + 8 weeks**
**Medication Status**	**On**	**Off**	**Off**	**Off**	**Off**	**Off**
UPDRS motor subscale	X	X	X	X	X	X
Beck Depression Inventory II		X		X		
University of Pennsylvania Smell Identification Test	X	X		X	X	
Timed Up and Go	X	X	X	X	X	X
Hopkins Verbal Learning Test	X	X	X	X	X	X
Parkinson’s Disease Questionnaire-39		X		X		
Trail Making Test	X	X	X	X	X	X
Simple & Choice Reaction Task	X	X	X	X	X	X
Processing Speed Test	X	X	X	X	X	X
Postural Sway	X	X	X	X	X	X
Nine Hole Peg Test	X	X	X	X	X	X
MRI	X	X		X	X	

* Baseline ‘on meds’ and ‘off meds’ are randomly assigned to be testing day 1 or 2.

Clinical and biomechanical measures will be used to assess motor and non-motor outcomes. The primary clinical motor outcome measure will be the UPDRS motor subscale, a well-studied test used to obtain a comprehensive clinical rating of PD [22]. The rater for this test will be blind to subject randomization. A secondary motor outcome will be the Timed Up and Go (TUG) is a reliable physical performance measure that assesses the time required for an individual to stand from a chair, ambulate 3 meters, turn 180 degrees, ambulate back to the chair, and return to a seated position [30].

Other clinical non-motor assessments will include the Parkinson’s Disease Questionnaire-39 (PDQ-39), University of Pennsylvania Smell Identification Test (UPSIT), Hopkins Verbal Learning Test, and the Beck Depression Inventory II (BDI-II). The PDQ-39 is a 39-item questionnaire that assesses the quality of life in individuals with PD [31]. In the UPSIT, which is used to assess hyposmia, the participant is asked to identify 40 odorants presented on microencapsulated booklet [32]. The Hopkins Verbal Learning Test is used to assess verbal memory of the participant [33], and the BDI- II questionnaire assesses depressive symptomology [34].

In an effort to gather biomechanical outcomes, several novel electronic mobile applications will be utilized. The modules, developed on the Apple iPad, include the Trail Making Test, Simple Reaction Task, Choice Reaction Task, Processing Speed Test, and a measure of postural sway in standing. The utilization of the iPad modules to assess cognitive and motor function provides a method of obtaining biomechanical data in a cost and time efficient manner compared to traditional biomechanical measures. Briefly, the Trail Making Test module is based on a widely-used neuropsychological assessment designed to evaluate executive function that involves connecting numbers (1-2-3…) and a number/letter sequence (1-A-2-B….) that are arranged on paper [35]. In the case of the app, the targets are arranged in a similar manner while using Fitts’ Law to ensure the segment of difficulty is matched across the paper and pencil form compared to the iPad version [36]. The electronic version administered on the iPad samples the time and X-Y position data at 60Hz which is then used to determine when the patient is moving versus searching for the next target [37]. The Simple Reaction Task module provides quantitative data on information processing by measuring the time between the presentation of a stimulus and the participant’s response [37]. The Choice Reaction Task module measures reaction time with more complex thinking, as one of two stimuli is randomly activated and the participant is timed from presentation to selection of the stimuli [37]. The technology of the iPad allows the reaction time speed to be measured accurately to the millisecond. The Processing Speed Test module is a modified, electronic version of the paper-and-pencil symbol-digit matching test which assesses complex attention and information processing speed [38,39]. For the module, the participant is presented with nine symbols matched with Arabic numerals 1-9. Below the key, symbols alone are presented to the participant. The participant is asked to correctly match as many symbols as possible to the corresponding numeral in 90 seconds.

**Figure 2:**
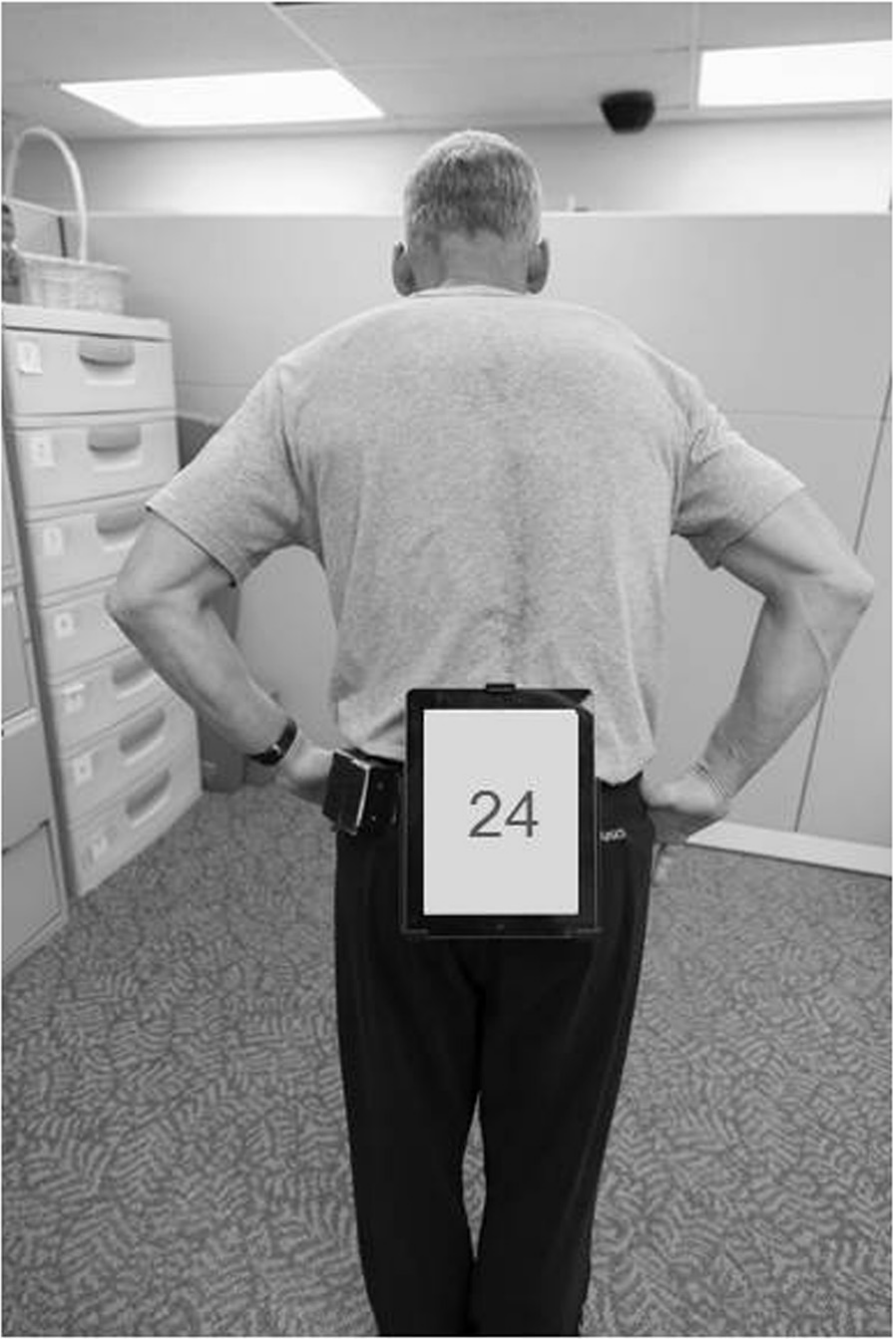
The iPad’s gyroscope and accelerometer capture linear and rotational movement to calculate volume of postural sway when belted to the waist of the participant. In this figure, the iPad is counting down from 30 seconds as the participant performs the following stances: 1) double leg stance, firm surface, eyes open; 2) double leg stance, firm surface, eyes closed.

As a biomechanical measure of balance, the gyroscope and accelerometer of the iPad will measure volume of postural sway [37,40-43]. We have validated the use of the iPad to characterize balance in young, healthy older adults, and individuals with PD [37,40-43]. Attached to the waist of the participant, the iPad captures linear and rotational movement to calculate the volume of postural sway (Figure 2). The postural sway test consists of a 30-second trial on the following conditions: 1) firm surface, double leg stance, eyes open; 2) firm surface, double leg stance, eyes closed.

**Figure 3:**
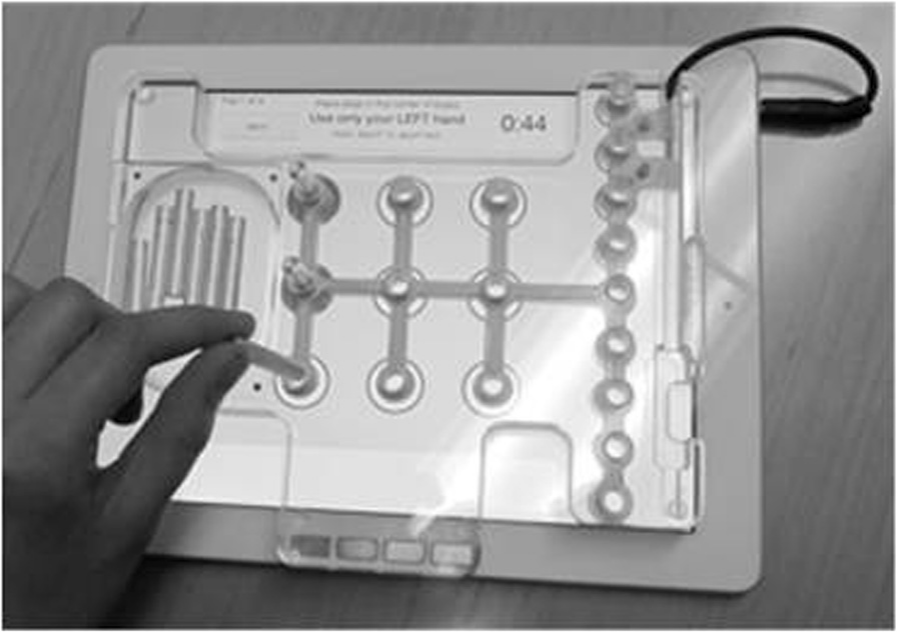
A transparent plastic overlay attaches to the iPad to create the electronic version of the NHPT. The participant performs the first segment of the NHPT by transferring the pegs from a dish to one of the 9 holes, removing the pegs, and returning them to the dish, which is measure of in-hand manipulation and manual dexterity. The second part of the test involves transferring pegs to and from a row (pictured on the right of the iPad screen below) rather than the dish to calculate transport time only. The capabilities of the iPad allows for detection of the time between each peg insertion and removal.

The Nine Hole Peg Test (NHPT) will be used as a measure of manual dexterity [44]. Consistent with our effort to objectify our outcome measures, the NHPT module is an electronic form of the traditional NHPT (Figure 3). Participants are instructed to pick up individual pegs from a dish, place them in the nine peg slots until all slots are filled, and then remove the pegs. The electronic version is an overlay mold over the iPad and has been shown to be reliable in individuals with multiple sclerosis [39]. The iPad capacitive touch screen detects the time between each peg insertion and removal, which provides a more precise temporal-spatial understanding of manual dexterity than the traditional measure.

### MRI data collection

For this study, structural MRI, functional MRI (fMRI), fcMRI and cerebral blood flow (CBF) data will be acquired on four occasions: Baseline (on and off antiparkinsonian medication), EOT, and EOT + 4 weeks. The structural scan is a T1-weighted anatomic image for identifying brain anatomy. The fMRI and fcMRI scans will be acquired with blood oxygen level-dependent (BOLD)-weighted echoplanar imaging scans. Two fcMRI scans will be acquired while subjects rest in the scanner with eyes closed. Two fMRI scans will be acquired in each session while subjects complete finger tapping in the following self-paced repeating sequence: digit 1, digit 3, digit 5, digit 2, digit 4 with each limb (left and right limbs, in random order) in a block paradigm (alternating 48 second-long blocks of tap and rest phases, repeated 4.5 times) to determine the relationship between changes in upper extremity motor performance and pattern of brain activation. Additional measures recorded for exploratory purposes include fcMRI between nodes of the putative motor network.

### Data analysis

Participants in all three groups will be compared descriptively on potentially confounding baseline variables (i.e., age, disease severity, and levodopa equivalent daily dose (LEDD)) to assess the extent of any imbalance across groups. Baseline variables in which there appears to be a clinically important baseline difference, or in which the standardized difference (absolute value of difference in means divided by pooled SD) between any 2 groups is greater than 10% will be included as covariates. P-values from these baseline variable comparisons will not be used to determine the need for covariate adjustment, as that criterion can be misleading.

The FE, VE, and the no-exercise control groups will be compared on each outcome of interest (motor, non-motor) using repeated measures analysis of covariance. For motor and non-motor outcomes, groups will be compared on outcomes at the mid-treatment, EOT, EOT + 4 weeks, and EOT + 8 weeks adjusting for the baseline (on and off medication) period as a covariate. The effects of group, time, and the group-by-time interaction will be assessed for each outcome. In the case of a significant interaction, the groups will be compared at each time point. Tukey’s correction for multiple comparisons will be used. Data will be transformed as needed to meet model assumptions. In addition to p-values, the estimated treatment effect and its 95% CI will be of interest as these data will aid in formulating exercise recommendations and potential benefits. For each hypothesis, significance level will be set at 0.05. Each participant’s change in fitness based on change in peak VO_2_ from CPX testing will be used as a covariate. This will remove the effect of possible differences in improvement in fitness level across the groups from confounding the results. Correlation between LEDD and the time spent within target HR zone during training, amount of work performed, and change in primary outcome variables in motor and non-motor outcomes will be assessed. If the LEDD is significantly correlated with these outcomes, it will be included as a covariate in the related analysis.

### MRI data

Functional imaging data collected during the motor tasks will be retrospectively motion corrected with a slice-based second-order motion model [45,46] in parallel with physiologic noise regression [47], spatially filtered [48] and analyzed for task-related activation using a least-squares fit of the voxel-level BOLD signal to a reference function generated from the task design [49]. The result will be a whole brain map of Student’s t-values reflecting the response of each voxel for each task. A paired *t*-test of the total volume of activation above a t-value of 3.5 (p < 0.001, uncorrected) in the supplemental motor area will be calculated in all subjects. Correlation will be calculated between the change in activation volume of the supplemental motor area and subcortical structures of interest (globus pallidus, putamen, and thalamus) for each subject and the corresponding biomechanical measures during a finger tapping task during the scan and UPDRS rating.

### Arterial spin labeling data and CBF measurement

Exercise may have an effect on baseline CBF. Basal CBF will affect the amplitude of the BOLD signal in response to a given neuronal activation level. In order to control for this possible confound to our measurement, we will measure basal CBF during each imaging session in order to 1) assess whether there are observed baseline CBF changes with the proposed exercise regimen, and 2) to permit the inclusion of this measure in a population analysis in order to control for this effect. A Q2TIPS arterial spin labeling (ASL) scan will be used to measure baseline CBF [50]. The ASL scan parameters are as follows: TE/TR/Flip = 2300/13/80, 12 axial slices 5mm thick, 1mm slice gap, FOV = 256x256, matrix = 64x64, TI1/TI2/SST = 700/1400/1100, bandwidth = 150kHz, repetitions = 139, Tag Slab = 100mm with 25mm gap.

### Adverse events

Serious adverse events, defined as an adverse experience that results in death, life-threatening experience, hospitalization, or significant disability, will be reported to the Cleveland Clinic Institutional Review Board within 10 working days. Non-serious adverse events will be reported annually. At the time of any adverse event, the event will be formally recorded by a member of the study team and reviewed by the primary investigator, who will determine if protocol or informed consent changes are necessary.

## Discussion

Although exercise prescription has become an increasing popular treatment for individuals with PD, there are currently no specific recommendations regarding mode, intensity, and duration [9]. In this protocol, we are prescribing a very specific mode, intensity, and duration of exercise with the hope of providing insight about exercise prescription. Specifically with aerobic exercise, it has been noted that there are gaps in literature on the immediate and long-term effects on PD symptoms [15]. Our follow-up periods include assessments up to 8 weeks following cessation of the intervention to determine the interim effects of this form of exercise.

While the UPDRS is a readily accepted outcome measure with PD; there is a lack of biomechanical outcome measures used in clinical trials for neurological populations. Through literature review, previous experience, and knowledge of the disease symptomology, the selected outcomes are comprehensive, objective, and systematic which will provide the opportunity to truly understand the clinical and behavioral effects of FE and VE on PD function. By automating several outcome measures to provide a more detailed and precise data collection tool, there is the potential for further understanding of PD symptomology. For example, in the Trail Making Test, the movement and dwell time can be separated, thus allowing the investigators to determine if time spent on a test is due to movement time, indicating a motor impairment, or dwell time, indicating a cognitive impairment from the individual processing which digit or number is next in the sequence. Likewise with the iPad version of the NHPT, the participant performs 2 different versions of the test (Figure 3). As a measure of in-hand manipulation, the first version consists of having the participant grasp, manipulate, and transfer the pegs from a dish to one of the 9 holes, remove the pegs, and return them to the dish. The second part of the test involves transferring pegs to and from a row, rather than the dish, to calculate transport time only. This differentiation between in-hand manipulation and transfer time could be helpful in differentiating between deficits.

Several of these iPad modules, including the postural sway test, NHPT, and Processing Speed Test, have been found to be reliable, sensitive, and clinically meaningful in individuals with multiple sclerosis [39]. This method of standardized testing, if found to be reliable and feasible in individuals with PD, will provide further evidence that objective, quantitative outcomes that can be used in both a research and a clinical setting are adventitious for neurological populations.

CYCLE Trial has the potential to make a unique contribution through the neuroimaging component. We have published researched that supports the benefit of a one-time bout of cycling on motor cortex functional connectivity; [23] however, the long-term effects of exercise on CNS connectivity is not known. By conducting imaging in various on and off-medication states at baseline, EOT, and EOT + 4 weeks, this study will provide insight into the potential neurophysiological CNS changes that exercise can produce in individuals with PD.

Overall we feel that the results of this study have the potential to directly impact patient care and will add to a body of evidence that challenges clinicians to view exercise as medicine for individuals with PD.

## Abbreviations

PD: Parkinson’s disease
CNS: Central nervous system
FE: Forced exercise
VE: Voluntary exercise
UPDRS: Unified Parkinson’s Disease Rating Scale
fcMRI: Functional connectivity Magnetic Resonance Imaging
CYCLE: Cyclical Lower Extremity Exercise for Parkinson’s disease
CI: Confidence interval
EOT: End of treatment
SD: Standard deviation
CPX: Cardiopulmonary exercise testing
HR: Heart rate
MRI: Magnetic Resonance Imaging
PDQ-39: Parkinson’s Disease Questionnaire-39
UPSIT: University of Pennsylvania Smell Identification Test
BDI-II: Beck Depression Inventory II
NHPT: Nine Hole Peg Test
fMRI: Functional Magnetic Resonance Imaging
CBF: Cerebral blood flow
BOLD: Blood oxygen level-dependent LEDD, levodopa equivalent daily dose
VO_2_: Volume of oxygen uptake
ALS: Arterial spin labeling
